# Effect of body mass index on duration of total knee replacement surgery: A prospective cross sectional study

**DOI:** 10.1016/j.amsu.2022.104637

**Published:** 2022-09-23

**Authors:** Zamin Abbas, Sohail Hafeez, Ali Naseem, Yasir Habib, Hassan Mumtaz

**Affiliations:** a Shifa International Hospital, Islamabad, Pakistan; b Health Services Academy, Islamabad, Pakistan

**Keywords:** TKR, Quality of life, BMI

## Abstract

**Introduction:**

Obesity and increased BMI has raised concerns throughout the globe. As obesity is often associated with many serious medical conditions. Obesity, older age and gender are major contributing factors for knee replacement surgeries. We aimed to compare the mean duration of surgery in obese and non-obese patients undergoing total knee replacement.

**Methods:**

A Cross-sectional study is conducted at the orthopedic department at Shifa international hospital, Islamabad during June 2021–Dec 2021. Study is conducted to assess the effect of BMI on duration of total knee arthroplasty. Sample size was calculated to be 105 with 95% confidence limit. Data will be analyzed using SPSS version 22. Quantitative variables like age, BMI and duration of surgery were presented as mean and standard deviation. Qualitative variables like gender, laterality (unilateral/bilateral), and ASA were presented as frequency and percentage.

**Results:**

There were more females undergoing the procedure than males. The predominant age group was found to be 56–65 years. On BMI classification scale, a far greater number of individuals were found to be obese constituting more than ⅗ of the study population and almost ⅕ of the patients were overweight. The Association of BMI Classification & Duration of Surgery has a significant p value of 0.00.

**Conclusion:**

A linear and direct relation was observed between body mass index and duration of surgery. There may be other contributing factors and will need more data and research.

## Highlights

• The body mass index (BMI) is widely recognized as a prognostic factor in multiple operations.
• High body mass index (BMI) is associated with increased rates of complications after total knee arthroplasty.
• Our study finds a strong association of Age, Gender & ASA Grade with BMI.

## 1 Introduction

There are contradictory reports of outcome of total knee replacement surgery in obese patients. Worldwide there has been an increase in the number of people suffering from osteoarthritis in combination with obesity. To ease pain and improve functional status of osteoarthritis patients, the most effective treatment is joint replacement. But obesity in this picture has altered the effectiveness of total knee replacement [[1], [2], [3]].

Body Mass Index is calculated as mass in kg/height in meters squared. WHO categorizes the BMI into six categories (underweight, normal, pre obesity/overweight, obesity class 1, obesity class 2 and obesity class 3). Joint replacement performed in patients with body mass index (BMI) > 30 kg/m^2^ has shown good results with decreased pain and improvement in quality of life [4]. However, these patients are prone to develop obesity related pre and postoperative complications [5].

Surgery in obese patients is technically challenging leading to reluctance of the orthopedic surgeons to perform an elective joint replacement in patients with a high BMI, particularly >40 kg/m^2^ [6]. Operative time is thought to be higher in obese patients.

Studies have shown that increased BMI can lead to increased duration of surgery that may need additional anesthesia or analgesia resulting in overall increased risk of infection and higher cost of the procedure. However, the studies done in this regard are few and non-conclusive, except one study (quoted above), which showed that the duration of surgery was more in obese patients, although the statistical difference was insignificant. Moreover, there is no local study on this subject. Rationale of this study is to compare the mean duration of surgery in obese and non-obese patients undergoing total knee replacement.

## 2 Methods

We conducted a cross-sectional study at the orthopedic department of Shifa International Hospital, Islamabad during June 2021–Dec 2021 to assess the effect of BMI on the duration of total knee arthroplasty surgery. Sample size was calculated to be 105 with 95% confidence levels and 8% margin for error, taking expected obesity i.e., 77.5% in patients undergoing total knee replacement.

Both males and females of ages ranging from 45 to 70 years opting for total knee replacement were included. On the other hand, patients with ASA III and IV, having underlying conditions like rheumatoid arthritis, Osteomalacia or metastasis of bone and patients undergoing revision surgery were excluded. Informed consent was taken and demographics (name, age, gender, laterality, BMI and ASA) were noted. Then patients will undergo surgery by a single surgical team (to prevent bias) as per standard method with assistant of researcher. Duration was measured in minutes from the time of incision till closure.

Our study is in accordance with the STROCSS 2021 guidelines [7]. A detailed STROCSS 2021 check list may be found in the supplemental materials. Our study is registered in Research Registry UIN researchregistry8066 [8]. Our research adheres to the principles outlined in the Helsinki Declaration. Ethical approval was given by Shifa International Hospital.

Data will be analyzed using SPSS version 22. Quantitative variables like age, BMI and duration of surgery were presented as mean and standard deviation. Qualitative variables like gender, laterality (unilateral/bilateral), and ASA were presented as frequency and percentage. Chi square tests were applied to see the association. *P*-value ≤0.05 were considered as significant.

## 3 Results

Data of 105 patients which complied with the inclusion criteria were included in the study, out of which females were predominant with more than ¾ of the total numbers. Ages were distributed among different groups with a 10 year difference and half of the population comprised the age group between 56 and 65 years., as shown in Table 1.

**Table 1:** Showing gender & age characteristics.

	Frequency	Percentage
**Gender**		
Male	11	10.5
Female	94	89.5
**Age**		
45–55 years	15	14.3
56–65 years	52	49.6
66–70 years	38	36.1

When the data was analyzed thoroughly the side of the injury section almost had a tie between unilateral and bilateral with bilateral having 5 more cases. ASA assessment was done, slight risk patients were greater in numbers i. e half of the study population lied in grade 2 category. No severe risk patients were seen while conducting the study, as shown in Table 2.

**Table 2:** Showing side of the injury & ASA grade.

	Frequency	Percent
**Side of the Injury**		
Bilateral	50	47.6
Unilateral	55	52.4
**ASA Grade**		
Grade 1	24	22.86
Grade 2	81	77.14

On BMI classification scale, a far greater number of individuals were found to be obese constituting more than ⅗ of the study population and almost ⅕ of the patients were overweight. To assess the hypothesized relationship between BMI and duration of surgery, time duration of each surgery was noted and statistics showcased that more than half of the population took more than 90 min in the OR. The Association of BMI Classification & Duration of Surgery has a significant p value of 0.00, as shown in Table 3.

**Table 3:** Showing association of BMI classification & duration of surgery.

		BMI Classification			Total	*P*-value
		Normal	Obese	Overweight		
Duration of Surgery	45 mins-1 hr.	11	0	0	11	0.00
	1 h −1 h 15 min		0	19	19	
	1 h 15 mins −1 h 30 mins	0	75	0	75	
Total		11	75	19	105	

The correlation between gender and BMI, age and BMI, side of injury and BMI, ASA grade and BMI, and gender with surgery duration is found to have a significant p value of 0.00, indicating a statistically significant relationship, as shown in Table 4.

**Table 4:** Showing the associations of different variables.

Association	Value	Difference	P Value
Gender with BMI	105.000^a^	2	.000
Age with BMI	129.640^a^	6	.000
Side of the Injury with BMI	29.950^a^	2	.000
ASA Grade with BMI	65.890^a^	4	.000
Gender with Duration of Surgery	60.286^a^	3	.000

## 4 Discussion

There has been an increasing trend in weight gain worldwide, obesity is linked with multiple medical conditions like cardiovascular disorders, diabetes and arthritis etc [9]. Kristen et al. reported that risk of knee replacement surgery is increased by more than 40% in obese or overweight individuals [10].

A detailed analysis of the results revealed that surgical duration of both unilateral and bilateral knee replacement had a linear and direct relationship with the BMI which is in synchrony with the figures depicted by Ref. [11]. For a unilateral knee replacement of a normal or lean person took almost an hour (60 min). While, for obese individuals it took almost one and a half hour (90 min). In addition, the time noted for the bilateral knee replacements were almost double than the unilateral ones. On the other hand, lozano et al. found no such differences in operative duration between normal and obese patients [12]. Our study found out that duration of surgery was far less in males than females (p = 5.10 × 10^-13) and this finding in synchrony with the results that the greatest number of obese patients were females further endorsing the relationship between BMI and duration of surgery.

Valderrama et al. reported that there was no relationship between BMI and early complications of Total knee replacement (TKR) [13]. A review study conducted by Sundaram et al. supported and backed the findings, and stated that a greater BMI doesn't make patients susceptible to early complications [14]. A prospective cohort study identified that obesity doesn't significantly influence a patient's functional abilities and pain [15].

This study highlighted that obesity is more prevalent in females in Pakistan, and a study in Libya also reported similar results [16]. Consequently, there are more female patients going through total knee replacement surgery as observed in this study and supported by research conducted by Keenan et al. [17].

Research data has identified that obesity does play a role or contributes to the length of time a patient stays in a hospital before getting discharged [18]. And as number of days increases the cost of the procedure also adds up increasing the overall expense for obese patients [19]. Furthermore, to conclude obesity is becoming a concern worldwide owing to being the root cause of many conditions, and obesity, age and gender are the major factors leading to total knee replacement.

Our study found out that duration of surgery was far less in males than females (p = 5.10 × 10^-13) and this finding in synchrony with the results that the greatest number of obese patients were females further endorsing the relationship between BMI and duration of surgery.

### 4.1 Limitations & strengths

Small sample size was a limitation to our study and some patients lost to follow-up. Our study finds strong association of Age, Gender & ASA Grade with BMI.

## 5 Conclusion

Though a detailed description about the topic is written above, to sum up the whole picture a fair judgement would be that obesity or BMI index does play a role or have an impact on the surgical duration of patients undergoing TKR, and a linear direct relationship in witnessed between BMI and duration of surgery.

## Provenance and peer review

Not commissioned, externally peer-reviewed.

## Ethical approval

Ethical approval granted by Shifa International Hospital, ref no IRB-116-21 dated June 06, 2021.

## Sources of funding

Not applicable.

## Author contributions

1. The main concept was determined by Sohail Hafeez.

2. Collection of data is done by Ali Naseem.

3. Data is analyzed and interpreted by Hassan Mumtaz.

4. Writing of the manuscript is done by Zamin Abbas.

5. Manuscript editing is done by Yasir Habib.

## Trial register number

1. Name of the registry: Research Registry
2. Unique Identifying number or registration ID: researchregistry8066
3. Hyperlink to your specific registration (must be publicly accessible and will be checked): N=Browse the Registry - Research Registry

## Guarantor

Not applicable.

## Consent

Not applicable.

## Declaration of competing interest

There is No conflict of interest.
